# A Confidence Interval for the Wallace Coefficient of Concordance and Its Application to Microbial Typing Methods

**DOI:** 10.1371/journal.pone.0003696

**Published:** 2008-11-11

**Authors:** Francisco R. Pinto, José Melo-Cristino, Mário Ramirez

**Affiliations:** 1 Instituto de Microbiologia, Instituto de Medicina Molecular, Faculdade de Medicina de Lisboa, Lisboa, Portugal; 2 Centro de Química e Bioquímica, Departamento de Química e Bioquímica, Faculdade de Ciências da Universidade de Lisboa, Lisboa, Portugal; University of East Piedmont, Italy

## Abstract

Very diverse research fields frequently deal with the analysis of multiple clustering results, which should imply an objective detection of overlaps and divergences between the formed groupings. The congruence between these multiple results can be quantified by clustering comparison measures such as the Wallace coefficient (W). Since the measured congruence is dependent on the particular sample taken from the population, there is variability in the estimated values relatively to those of the true population. In the present work we propose the use of a confidence interval (CI) to account for this variability when W is used. The CI analytical formula is derived assuming a Gaussian sampling distribution and recurring to the algebraic relationship between W and the Simpson's index of diversity. This relationship also allows the estimation of the expected Wallace value under the assumption of independence of classifications. We evaluated the CI performance using simulated and published microbial typing data sets. The simulations showed that the CI has the desired 95% coverage when the W is greater than 0.5. This behaviour is robust to changes in cluster number, cluster size distributions and sample size. The analysis of the published data sets demonstrated the usefulness of the new CI by objectively validating some of the previous interpretations, while showing that other conclusions lacked statistical support.

## Introduction

Clustering is frequently used to analyze data in many diverse fields such as the life and medical sciences, computer sciences, social sciences, economics and engineering. There are many different approaches to clustering and one may use different sets of individual characteristics to generate different classifications or clusterings. In face of two different clusterings of the same set of individuals, one can measure the extension of agreement between them and, if the results are in agreement, it may be enough to collect data from a single source. On the other hand, if the two clusterings disagree, combining their results may offer additional information and discriminatory power. Researchers in such diverse fields as bioinformatics [1], computer science [2], psychology [3] and ecology [4], have developed and applied methods to compare clusterings. While some methods provide a global measure of concordance between clusterings [3], that may also take into account inter-cluster distances [1], others offer an asymmetric view of concordance in which the agreement of clustering A with B may be different of the agreement of B with A [5]. One of the latter methods is the Wallace coefficient (W), which has recently been successfully applied to the analysis of microbial typing data [6].

Microbial typing methods provide clinical microbiologists with a fundamental tool for the epidemiological characterization of microbial pathogens by allowing the distinction of diverse organisms of the same species. These tools have been used to identify particularly virulent strains, to measure their spread between hosts and in general to clarify the evolutionary history and population dynamics of microbial pathogens. A variety of typing methods are available, targeting different phenotypic or genotypic properties of microbial isolates. To be able to compare or combine studies performed using different methods, it is important to know if the various methods are identifying the same relationships between strains. In other words, it is important to determine the degree of congruence between the resulting clusterings. A common framework for comparing and relating multiple typing methods with objective measures has been proposed [6] and applied in several subsequent studies to a diverse array of typing techniques in different bacterial species [7], [8], [9], [10], [11], [12]. An online tool has been developed to allow the easy application of these measures by the microbial typing community (www.comparingpartitions.info) and scripts for the popular Bionumerics software are also available [6].

One crucial analytical measure within this framework is W. Given two clusterings A and B, W of the classification provided by A to the classification provided by B is the probability that two individuals are classified together using method B knowing that they were classified together using method A. The intuitive interpretation of the values of W and their directionality has contributed to its successful use in microbial typing studies.

In spite of the value of W to quantitatively evaluate the congruence of the classifications of different clusterings, its use and interpretation could be improved with two lacking features: 1) estimation of the expected W value if the classifications are independent (W_i_) and 2) estimation of suitable confidence intervals for W. The first feature is important because even high values can be simply explained by chance agreement, or, reversely, low W values can be significantly higher than the value expected under the assumption of independence of classifications. The latter is a function of the number and relative size of clusters produced by each of the clusterings. Statistical confidence intervals are necessary to compare the W calculated between different clusterings because the obtained estimative of W can change with different samples of individuals. Since we are interested in the more general problem of quantifying the relationships between the classifications of different typing methods and not only in that particular set of individuals, i.e., we would like to estimate a population parameter using a given sample, confidence intervals are necessary to indicate the reliability of our estimate. Here we derive an analytical expression for the calculation of a CI for W and evaluate its performance in simulated and microbial typing data sets.

## Results and Discussion

### Derivation of CI expression

Consider a contingency table (*CT*) that contains the dual classification of each individual entity in both clusterings A and B. *CT* element *m_ij_* is the number of individuals belonging to both clusters *A_i_* and *B_j_*. *a_i_* is the sum of row *i* and *b_j_* is the sum of column j. W of A to B is defined as the ratio of the number of pairs of individuals with the same classification according to A and B over the number of pairs of individuals with the same classification according to A:
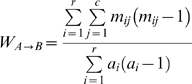
(1)For each row of CT we can compute the Simpson's index of diversity [13], [14] of the B clustering among elements of the *A_i_* cluster as:
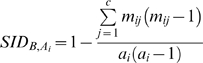
(2)The method to estimate both W_i_ and a confidence interval for W stems from the observation that the W of the classifications under method A to the classifications under method B is a weighted average of one minus the Simpson's index of diversity (1-SID) of the B classification within each cluster of A:
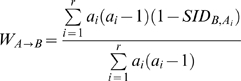
(3)If methods A and B produce independent classifications, it means SID*_B,Ai_* should be equal for each cluster *A_i_*. Consequently, the expected value of W when both classifications are independent is:
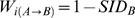
(4)Where SID*_B_* is the Simpson's index of Diversity of the B classification considering all studied individuals.

To assess if the estimated W is significantly different from the value expected under independence one could use a confidence interval. If the expected value is within the confidence interval boundaries, the null hypothesis of independence between classifications cannot be rejected with the respective confidence level. We deduced the confidence interval limits from the variance of SID, originally presented by Simpson [13]:
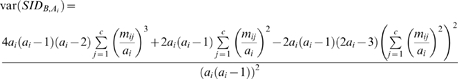
(5)Where *m_ij_* is the number of individuals belonging to both clusters *A_i_* and *B_j_*, and *c* is the number of B clusters. Considering the size of A clusters (*a_i_*) as constants and using three general properties of the variance of the variable X:
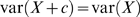
(6)

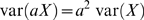
(7)Where *a* and *c* are constants, and if *X* and *Y* are independent variables:
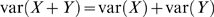
(8)We arrive at:
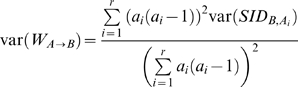
(9)Following the approach of Grundmann [14] we computed the confidence interval limits assuming a Gaussian distribution. Hence, for a 95% confidence interval, the limits are given by:
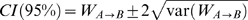
(10)


### Analysis of simulated clusterings

**Figure 1:**
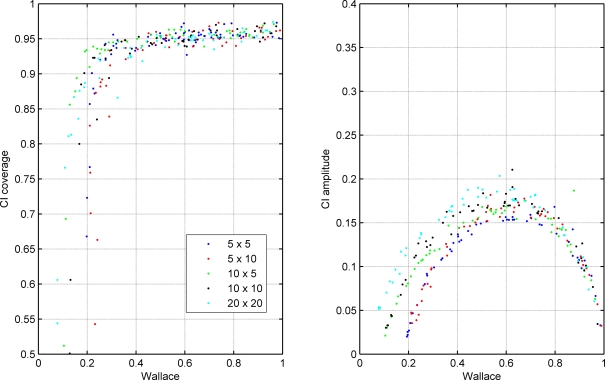
Coverage and amplitude of 95% confidence intervals for Wallace coefficient obtained from simulated classifications. Each dot represents a simulation with a particular set of parameters. The colors indicate the dimensions of the simulated contingency tables as indicated in the figure legend, which correspond to the number of clusters in each of the two classifications. All simulated tables in this plot had *n* = 300 elements and the distribution of row cluster sizes followed a Zipfian distribution with exponent *a* = 1.

**Figure 2:**
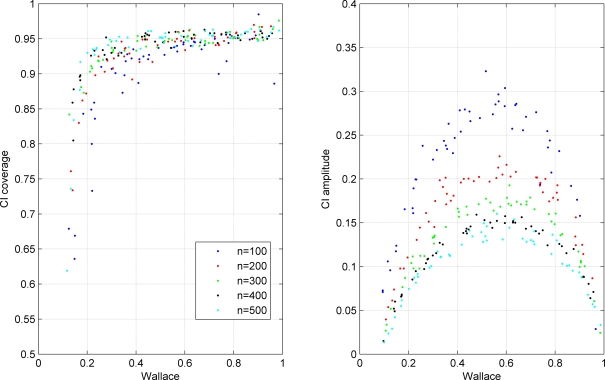
Coverage and amplitude of 95% confidence intervals for Wallace coefficient obtained from simulated classifications. Each dot represents a simulation with a particular set of parameters. The colors indicate the number of elements *n* of the simulated contingency tables as indicated in the figure legend. All simulated tables in this plot had 10×10 dimensions and the distribution of row cluster sizes followed a Zipfian distribution with exponent *a* = 1.

**Figure 3:**
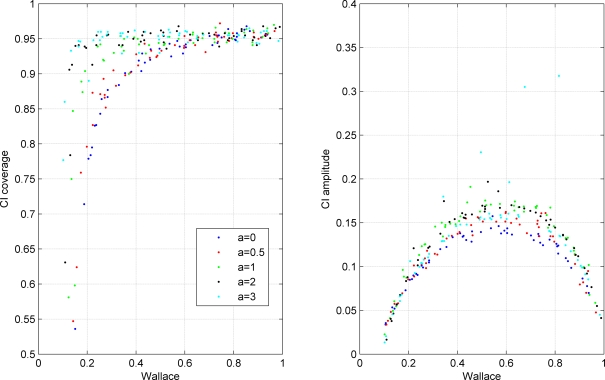
Coverage and amplitude of 95% confidence intervals for Wallace coefficient obtained from simulated classifications. Each dot represents a simulation with a particular set of parameters. The colors indicate exponent *a* of the Zipfian distribution determining the distribution of row cluster sizes of the simulated contingency tables as indicated in the figure legend. All simulated tables in this plot had *n* = 300 elements and 10×10 dimensions.

We validated the performance of this confidence interval using the simulation of random contingency tables representing the cross classification of two hypothetical clusterings. The results are presented in figures 1, 2, 3.

Analysis of figure 1 indicates that the proposed 95% confidence interval for W approximates the desired coverage of 95% when W is between 0.5 and 1. From 0.5 to 0, the coverage gradually decreases, meaning that the W sampling distribution is diverging from normality in this range. This behavior is quite robust to changes in the number of clusters in each of the two classifications. The change in table dimensions does not have a detectable impact on interval amplitude for high W values. For low W values, the interval amplitudes slightly increase for increasing row number. The curved shape of the amplitude as a function of W value resembles the relationship between the amplitude of a confidence interval for a simple proportion and the actual proportion value. Indeed, W can be seen as a proportion of individual pairs classified in the same cluster by both methods among the pairs classified in the same cluster by the first method.

In Figure 2 we studied the impact of sample size. The plot of interval coverage is similar to the one in Figure 1. For W values below 0.5 it is possible to observe that larger sample sizes have better coverage. There is also a clear impact of the number of elements in the amplitude of the resulting confidence intervals, with larger amplitudes for low sample sizes.

Finally, in Figure 3 we systematically change the distribution of row cluster sizes from a uniform distribution (*a* = 0) to a very skewed distribution where most of the elements are concentrated in a single cluster (*a* = 3). The distribution of cluster sizes normally found in microbial typing data, as well as in other biological data, is closer to a skewed scenario than to the uniform distribution. Interval amplitude is not significantly affected by these changes. The decrease in confidence interval coverage for low W values is stronger for cluster size distributions closer to uniformity.

Globally, the simulations performed show that the proposed confidence interval has the required 95% coverage for W values between 0.5 and 1 and non-uniform distribution of cluster sizes. This result is robust to changes in number of clusters, cluster size distribution and number of individuals sampled. The latter is the main parameter influencing the amplitude of the confidence intervals. The simulations also show that the confidence interval does not have the desired coverage for low W values (especially below 0.2) and this problem is more pronounced for uniform cluster size distributions. This should not be a major concern since, we are mainly interested in making comparisons between high W coefficients, and, as previously stated, most naturally occurring cluster size distributions in biology are not uniform. In this regard, it is important to note that the creation of maps of type equivalences proposed previously [6] would only be beneficial if the congruence between classifications would be high and an arbitrary, but reasonable, critical value would be W = 0.5. In the rare situations where it is important to know if a low W value is still significantly different from another value or from the independence hypothesis, the confidence interval presented in expression 10 is not appropriate. In the event that such situation is found, a possible approach would be to compute bootstrapped confidence intervals.

### Analysis of microbial typing data sets

To further demonstrate the usefulness of the proposed confidence interval, we applied it to three previously published data sets [6], [9], [10]. The results are presented in tables 1–5. These tables are similar to the ones presented in the original publications, but present two pieces of extra information: W_i_ for each column and the 95% confidence intervals for each estimated W. An analysis of these two values allows us to conclude if the information given by one typing method is independent or unrelated with the information given by the other method. We can reject this hypothesis (with 95% confidence) if the CI for that pair of methods does not include the W_i_ for the corresponding column in the table. For the typing methods and data sets explored here, independence is an exception that happens mainly when we probe the relationship between the classifications of a low diversity method with the classifications of others with very high diversities. This general dependence among typing methods supports the validity of the concept behind the use of typing techniques to infer relationships between microbial strains. The usual assumption confirmed here is that the differences found in a particular gene, genomic region or phenotype chosen for typing reflect in part the overall relationship between the strain genotypes. But it is important to clarify the difference between the absence of complete independence and the actual ability to predict the classification produced by a given method from the results obtained with another. For example, in the macrolide-resistant Lancefield group A streptococci (GAS) dataset [6] (Table 1), the W between T typing and Pulsed-Field Gel Electrophoresis (PFGE) type (of profiles resulting from digestion with either SmaI or Cfr9I endonucleases) (W_T→PFGE_ = 0.56 [0.48–0.64]) is significantly higher than the expected value under independence (0.19), but trying to predict PFGE type from T typing would lead to high error rates (from 36 to 52% of errors among strain pairs with the same T type), whereas the reverse (W_PFGE→T_) leads to much lower error rates (from 13 to 24%).

**Table 1:** Wallace coefficients and respective 95% confidence intervals for the methods used to characterize 325 macrolide-resistant GAS in (1).

	T typing	*emm* typing	SmaI/Cfr9I 80%	SfiI 68%	T+*emm*
W_i_ *	0.28	0.22	0.19	0.19	0.19
T typing		0.70 [0.62–0.77]	0.56 [0.48–0.64]	0.53 [0.45–0.61]	0.70 [0.62–0.77]
*emm* typing	0.86 [0.81–0.91]		0.80 [0.74–0.87]	0.72 [0.65–0.80]	0.86 [0.81–0.91]
SmaI/Cfr9I 80%	0.82 [0.76–0.87]	0.95 [0.93–0.97]		0.81 [0.74–0.88]	0.82 [0.76–0.87]
SfiI 68%	0.76 [0.71–0.82]	0.85 [0.80–0.90]	0.80 [0.74–0.86]		0.73 [0.67–0.79]
T+*emm*	1 [1–1]	1 [1–1]	0.80 [0.73–0.87]	0.72 [0.64–0.81]	

* Expected Wallace Coefficient if the classification of the method in the column is independent of the classifications of the methods in the rows.

**Table 2 pone-0003696-t002:** Wallace coefficients and respective 95% confidence intervals for the methods used to characterize 160 invasive GAS in (4).

	Toxin profile	T typing	*emm* typing	PFGE type
W_i_ *	0.18	0.12	0.08	0.08
Toxin profile		0.37 [0.31–0.44]	0.40 [0.34–0.47]	0.39 [0.33–0.44]
T typing	0.56 [0.47–0.66]		0.56 [0.46–0.66]	0.53 [0.43–0.62]
*emm* typing	0.90 [0.84–0.97]	0.83 [0.76–0.90]		0.89 [0.86–0.92]
PFGE type	0.87 [0.80–0.94]	0.78 [0.72–0.85]	0.89 [0.82–0.96]	

* Expected Wallace Coefficient if the classification of the method in the column is independent of the classifications of the methods in the rows.

**Table 3 pone-0003696-t003:** Wallace coefficients and respective 95% confidence intervals for the methods used to characterize 37 invasive GAS in (4) including MLST.

	Toxin profile	T typing	*emm* typing	PFGE type	ST
W_i_ *	0.21	0.10	0.04	0.05	0.04
Toxin profile		0.17 [0.08–0.26]	0.19 [0.10–0.28]	0.16 [0.08–0.24]	0.17 [0.08–0.27]
T typing	0.37 [0.24–0.50]		0.28 [0.18–0.38]	0.2 [0.07–0.33]	0.28 [0.18–0.38]
*emm* typing	0.90 [0.79–1]	0.60 [0.44–0.76]		0.57 [0.40–0.73]	0.7 [0.57–0.82]
PFGE type	0.67 [0.52–0.81]	0.39 [0.22–0.57]	0.52 [0.34–0.69]		0.48 [0.33–0.64]
ST	1 [1–1]	0.75 [0.60–0.90]	0.88 [0.74–1]	0.67 [0.49–0.84]	

* Expected Wallace Coefficient if the classification of the method in the column is independent of the classifications of the methods in the rows.

**Table 4 pone-0003696-t004:** Wallace coefficients and respective 95% confidence intervals for the methods used to characterize 116 MRSA in (3).

	PFGE type	PFGE subtype	*spa* type	BURP	ST	eBURST	*SCC_mec_*	PFGE type +*spa* type	PFGE subtype +*spa* type	PFGE type+*SCC_mec_*	PFGE subtype+*SCC_mec_*	*SCC_mec_*+ST
W_i_ *	0.06	0.005	0.04	0.21	0.09	0.29	0.17	0.02	0.003	0.03	0.004	0.04
PFGE type		0.09 [0.03–0.14]	0.29 [0.19–0.39]	0.83 [0.74–0.92]	0.32 [0.24–0.40]	0.82 [0.75–0.90]	0.47 [0.37–0.57]	0.29 [0.19–0.39]	0.06 [0–0.11]	0.47 [0.37–0.56]	0.08 [0.02–0.13]	0.24 [0.16–0.31]
PFGE subtype	1 [1–1]		0.67 [0.55–0.78]	0.97 [0.94–1]	0.82 [0.73–0.90]	1 [1–1]	0.88 [0.82–0.94]	0.67 [0.55–0.78]	0.67 [0.55–0.78]	0.88 [0.82–0.94]	0.88 [0.82–0.94]	0.76 [0.66–0.85]
*spa* type	0.40 [0.30–0.51]	0.08 [0.02–0.14]		1 [1–1]	0.60 [0.46–0.74]	0.96 [0.94–0.98]	0.54 [0.42–0.66]	0.40 [0.30–0.51]	0.08 [0.02–0.14]	0.28 [0.18–0.39]	0.07 [0.01–0.13]	0.38 [0.25–0.51]
BURP	0.22 [0.17–0.27]	0.02 [0–0.04]	0.19 [0.13–0.26]		0.39 [0.29–0.49]	0.76 [0.68–0.85]	0.28 [0.23–0.34]	0.08 [0.04–0.11]	0.02 [0–0.03]	0.12 [0.07–0.16]	0.02 [0–0.04]	0.16 [0.11–0.21]
ST	0.21 [0.15–0.27]	0.05 [0.02–0.08]	0.29 [0.20–0.38]	0.96 [0.90–1]		1 [1–1]	0.41 [0.33–0.50]	0.12 [0.07–0.17]	0.03 [0–0.06]	0.16 [0.10–0.21]	0.04 [0.01–0.07]	0.41 [0.33–0.50]
e-BURST	0.16 [0.12–0.20]	0.02 [0–0.03]	0.14 [0.09–0.18]	0.56 [0.50–0.62]	0.30 [0.22–0.37]		0.20 [0.16–0.24]	0.05 [0.02–0.08]	0.01 [0–0.02]	0.08 [0.04–0.11]	0.01 [0–0.03]	0.12 [0.08–0.16]
SCC_mec_	0.16 [0.11–0.20]	0.02 [0–0.05]	0.13 [0.08–0.18]	0.36 [0.29–0.43]	0.21 [0.15–0.27]	0.35 [0.28–0.42]		0.07 [0.03–0.11]	0.02 [0–0.04]	0.16 [0.11–0.20]	0.02 [0–0.05]	0.21 [0.15–0.27]
PFGE type +*spa* type	1 [1–1]	0.20 [0.07–0.32]	1 [1–1]	1 [1–1]	0.62 [0.46–0.79]	0.94 [0.90–0.97]	0.70 [0.56–0.85]		0.20 [0.07–0.32]	0.70 [0.56–0.85]	0.18 [0.06–0.30]	0.53 [0.36–0.69]
PFGE subtype +*spa* type	1 [1–1]	1 [1–1]	1 [1–1]	1 [1–1]	0.86 [0.77–0.96]	1 [1–1]	0.91 [0.84–0.97]	1 [1–1]		0.91 [0.84–0.97]	0.91 [0.84–0.97]	0.77 [0.66–0.89]
PFGE type+*SCC_me_*_c_	1 [1–1]	0.16 [0.07–0.25]	0.44 [0.32–0.56]	0.94 [0.87–1]	0.51 [0.39–0.63]	0.85 [0.81–0.90]	1 [1–1]	0.44 [0.32–0.56]	0.11 [0.03–0.20]		0.16 [0.07–0.25]	0.51 [0.39–0.63]
PFGE subtype+*SCC_mec_*	1 [1–1]	1 [1–1]	0.69 [0.57–0.81]	1 [1–1]	0.86 [0.78–0.94]	1 [1–1]	1 [1–1]	0.69 [0.57–0.81]	0.69 [0.57–0.81]	1 [1–1]		0.86 [0.78–0.94]
*SCC_mec_*+ST	0.38 [0.29–0.47]	0.10 [0.04–0.17]	0.44 [0.33–0.56]	0.95 [0.90–1]	1 [1–1]	1 [1–1]	1 [1–1]	0.25 [0.16–0.33]	0.07 [0.01–0.13]	0.38 [0.29–0.47]	0.10 [0.04–0.17]	

* Expected Wallace Coefficient if the classification of the method in the column is independent of the classifications of the methods in the rows.

**Table 5:** Wallace coefficients and respective 95% confidence intervals for the methods used to characterize 82 MSSA in (3).

	PFGE type	PFGE subtype	*spa* type	BURP	ST	eBURST	PFGE type +*spa* type	PFGE subtype +*spa* type
W_i_ *	0.05	0.01	0.02	0.07	0.04	0.09	0.02	0.002
PFGE type		0.14 [0.06–0.22]	0.39 [0.24–0.54]	0.69 [0.54–0.84]	0.58 [0.45–0.72]	0.85 [0.73–0.98]	0.39 [0.24–0.54]	0.05 [0–0.11]
PFGE subtype	1 [1–1]		0.33 [0.15–0.52]	0.83 [0.75–0.91]	0.79 [0.68–0.90]	1 [1–1]	0.33 [0.15–0.52]	0.33 [0.15–0.52]
*spa* type	0.92 [0.88–0.95]	0.11 [0.01–0.21]		1 [1–1]	0.60 [0.42–0.78]	0.94 [0.91–0.98]	0.92 [0.88–0.95]	0.11 [0.01–0.21]
BURP	0.58 [0.44–0.72]	0.10 [0.03–0.17]	0.35 [0.21–0.50]		0.48 [0.39–0.59]	0.74 [0.64–0.85]	0.32 [0.18–0.47]	0.04 [0–0.09]
ST	0.79 [0.67–0.90]	0.15 [0.06–0.24]	0.34 [0.24–0.43]	0.78 [0.67–0.89]		1 [1–1]	0.32 [0.23–0.42]	0.06 [0–0.13]
e-BURST	0.48 [0.35–0.61]	0.08 [0.03–0.13]	0.22 [0.10–0.34]	0.50 [0.36–0.63]	0.42 [0.31–0.52]		0.22 [0.10–0.34]	0.03 [0–0.07]
PFGE type +*spa* type	1 [1–1]	0.12 [0.02–0.22]	1 [1–1]	1 [1–1]	0.62 [0.43–0.81]	1 [1–1]		0.12 [0.02–0.22]
PFGE subtype +*spa* type	1 [1–1]	1 [1–1]	1 [1–1]	1 [1–1]	0.88 [0.75–1]	1 [1–1]	1 [1–1]	

* Expected Wallace Coefficient if the classification of the method in the column is independent of the classifications of the methods in the rows.

The dataset of 160 invasive GAS isolates [9] is analyzed in Table 2 and the subset of 37 strains that where characterized by Multilocus Sequence Type (MLST) originated Table 3. The comparison of the two tables leads to two main observations. First, the confidence intervals are wider in table 3 as compared with table 2. This was expected and is explained by the lower number of strains analyzed in table 3. Second, most of the confidence intervals for corresponding W values in the two tables do not overlap, although all the strains used in table 3 were also in the set studied in table 2. This apparent contradiction reflects the fact that the 37 strains analyzed by MLST were not randomly selected. Only a few strains from each PFGE type were analyzed by MLST. This non-random selection of the sample artificially increased the diversity of PFGE types (and indirectly of other related typing methods) in the subset collection, resulting in negatively biased W values.

With the availability of W confidence intervals we can statistically validate some of the hypothesis posed in the original studies. In the macrolide-resistant GAS study [6] the authors state that PFGE types derived using profiles resulting from the digestion with SmaI/Cfr9I had higher predictive power over other methods when compared with the use of profiles resulting from the digestion with SfiI. In fact, the W of SmaI/Cfr9I PFGE type to *emm* type is significantly higher than from SfiI PFGE type (0.95 [0.93–0.97] and 0.85 [0.80–0.90] respectively). Although the trend is the same, the difference does not reach significance for the prediction of T type (0.82 [0.76–0.87] vs. 0.76 [0.71–0.82]) or T+*emm* types (0.82 [0.76–0.87] vs. 0.73 [0.67–0.79]). In the same study, the observation that PFGE type could predict *emm* type to a greater extent than the reverse is supported by non-overlapping 95% confidence intervals of W (0.95 [0.93–0.97] vs. 0.80 [0.74–0.87]). These results reinforce the importance of characterizing GAS using PFGE and the SmaI/Cfr9I endonucleases to define GAS clones.

Tables 4 and 5 refer to a comparison of typing methods applied to 116 Methicillin-Resistant *Staphylococcus aureus* (MRSA) and 82 Methicillin-Susceptible *Staphylococcus aureus* (MSSA) [10]. In the original publication, the authors discuss the differences in agreement between typing methods in the two populations. Among other comparisons, the confidence intervals confirmed that *spa* type was found to be a better predictor of PFGE type for MSSA (0.92 [0.88–0.95]) than for MRSA (0.40 [0.30–0.51]). Using only the point estimates of W the authors have concluded that both PFGE type and subtype were able to predict BURP group much better for MRSA (type: 0.83 [0.74–0.92]; subtype: 0.97 [0.94–1]) than MSSA (type: 0.69 [0.54–0.84]; subtype: 0.83 [0.75–0.91]) but this conclusion only has statistical support for the PFGE subtype level. For MRSA strains, they observed that the PFGE subtype-*spa* type combination performed better in the prediction of *SCC_mec_* type (0.91 [0.84–0.97]) than PFGE subtype alone (0.88 [0.82–0.94]), but again this difference is not statistically significant with 95% confidence. Similarly, the higher performance of PFGE type-*spa* type combination (0.71 [0.56 0.85]), as compared with each technique alone (PFGE type: 0.47 [0.37–0.57]; *spa* type: 0.54 [0.42–0.66]), in the prediction of *SCC_mec_* type was not statistically supported, indicating that there is no significant predictive power of *spa* type and PFGE type, either alone or in combination, in relation to *SCC_mec_* type. Regarding the prediction of eBURST group, we confirmed a better performance of PFGE type-*spa* type combination (0.94 [0.90–0.97]) relatively to the PFGE type-*SCC_mec_* type combination (0.85 [081–0.90]). On the other hand, we could not validate the higher predictive power of PFGE type-*SCC_mec_* type over BURP group (0.94 [0.87–1]) as compared to the prediction of eBURST group (0.85 [0.81–0.90]).

In spite of the data discussed above, that question some of the relationships between the results of typing methods discussed in the original publication, the availability of a CI for W confirmed the indication of using PFGE and *spa* typing as a cost-effective combination of techniques for a detailed characterization of *S. aureus* isolates.

The proposed 95% CI for W estimates allows a more powerful analysis of the correspondence between the classifications of typing methods. With this information in hand we can objectively detect if one method is recovering part of the information obtained from another method by comparing the confidence interval limits with the expected W value assuming independence between classifications (W_i_). The confidence intervals around the point estimate also allow the distinction of discrepancies in W values that are due to differences in the pattern of diversity of a given microbial population or that are only a consequence of sampling variability. Therefore, the availability of confidence intervals reinforces the important role of W in generating maps of type equivalences between typing methods. Such a tool allows not only for comparison of typing results obtained by different methods but also facilitates the joint analyses of multiple typing methods. The application of this approach to already published data, while confirming some of the prior interpretations based solely on W point estimates, did not lend statistical support to others that need further scrutiny. These findings strongly support both the necessity and the increased value of applying the proposed W confidence intervals, not only in microbial typing studies, but also in any field where comparison of clusterings can be used as a study tool.

## Methods

### Numerical simulations

We randomly generated classifications from two hypothetical methods A and B for sets of *n* individuals. This consisted in the construction of a two-way contingency table *CT* with *r* rows and *c* columns, meaning that method A produces *r* row clusters and method B produces *c* columns clusters. CT was generated according with the parameters *n* (sample size), *r* (number of rows), *c* (number of columns), *a* (parameter determining the distribution of cluster sizes) and *b* (parameter determining the approximate value of W).

Briefly, we generate the *r* cluster sizes obtained with method A according to a Zipfian distribution with exponent *a*. This means that if we rank the clusters by decreasing size, the number of elements in the cluster with rank *z* is proportional to *z^−a^*. Then, for each row cluster we randomly select a matching column cluster and allocate the row elements such that the probability of being assigned to the matching column cluster is *b*, and the probability of being assigned to any other cluster is *(1-b)/(c-1)*.

W coefficient from the row to column classifications and corresponding 95% confidence interval was computed from *CT* according to the expressions in the results section. Then, assuming that the counts in each row of the table follow a multinomial distribution, and using the row proportion of elements *CT* as the multinomial distribution population parameters, we randomly generated 1000 new tables *rCT_i_*. For each *rCT_i_* we estimated W from row to column classifications. We then calculated the confidence interval coverage as the fraction of W values of *rCT_i_* that were between the limits of the confidence interval computed from *CT*.

To evaluate the confidence interval performance under a range of different conditions, all the five parameters used to generate *CT* were systematically changed to produce multiple combinations of sample size, number of clusters, cluster size distribution and W range.
